# A survey of knowledge, attitudes and practices regarding malaria and bed nets on Bubaque Island, Guinea-Bissau

**DOI:** 10.1186/s12936-020-03469-1

**Published:** 2020-11-17

**Authors:** Harry Hutchins, Grace Power, Thomas Ant, Eunice Teixeira da Silva, Adriana Goncalves, Amabelia Rodrigues, James Logan, David Mabey, Anna Last

**Affiliations:** 1 London School of Hygiene and Tropical Medicine, Keppel Street, London, WC1E 7HT UK; 2 University of Glasgow, University Avenue, Glasgow, G12 8QQ UK; 3 Projecto de Saúde Bandim, Apartado, 8611004 Bissau Codex Guinea-Bissau

**Keywords:** Guinea-bissau, Malaria, Bed net, Household, Knowledge, Attitudes, Practices

## Abstract

**Background:**

Malaria remains a significant public health problem in Guinea-Bissau, West Africa. Government control measures include bed net distribution campaigns, however, local knowledge, attitudes and practices towards bed nets and malaria are uncharacterized on the remote Bijagos Archipelago.

**Methods:**

Knowledge, attitude and practice questionnaires were conducted with household heads, aiming to explore the understanding of malaria and factors influencing bed net uptake and usage. Nets were observed in situ to appraise net quality and behaviour. All 14 villages and one semi-urban neighbourhood on Bubaque Island were included. One in 5 households containing school-aged children were randomly selected.

**Results:**

Of 100 participants, 94 were aware of malaria and 66 of those considered it a significant or severe problem, primarily because of its impact on health and income. Transmission, symptoms and risk factors were well known, however, 28.0% of participants felt under-informed. Some 80.0% reported contact with distribution campaigns, with inter-village variability. Campaign contact was associated with feeling well informed (OR 3.44; P = 0.024) and inversely with perceiving malaria a household (OR 0.18; P = 0.002) or regional problem (OR 0.25; P = 0.018). Every household contained nets; every identifiable example was a long-lasting insecticide-treated net (LLIN), however, 23.0% of households contained at least one expired net. Replacements were in demand; 89.0% of households reported that all residents used nets, and average occupancy was 2.07 people per net; 65.2% stated that the repurposing of bed nets was common. Correctly using bed nets, defined by age, integrity and demonstration, was 35.0% and strongly associated with completing intermittent preventative treatment in pregnancy (RR 3.63; P = 0.014).

**Conclusions:**

Knowledge of malaria is good in these communities. Bed nets are used widely and are valued for their role in preventing malaria. However, their use is frequently sub-optimal and offers a target for improving malaria control by adapting popular distribution campaigns to provide more education alongside fresh LLINs. The impact of this could be significant as LLINs represent the mainstay of malaria prevention in Guinea-Bissau; however, the persistence of malaria despite the high uptake of LLINs seen in this study suggests that novel supplementary approaches must also be considered.

## Background

Malaria remains a significant public health issue in Guinea-Bissau, West Africa [1]. Whilst it is well-characterized in the capital, Bissau [2], published data from elsewhere in the country are scarce. Rural communities are known to hold different beliefs about malaria and typically exhibit a higher disease burden [3–9]. The Bijagos Archipelago, lying off the Atlantic coast, consists of 88 islands and islets, approximately 20 of which are inhabited by small permanent and semi-permanent populations living predominantly in forest villages supported by subsistence agriculture. The islands’ isolation presents significant challenges to healthcare provision, with no recent published epidemiological data to guide programmatic activities.

Government malaria control measures include enhanced case finding, intermittent preventive treatment in pregnancy (IPTp) and free triennial long-lasting insecticidal net (LLIN) distribution nationwide, most recently in May 2017. This latter intervention is at the core of national malaria control: it is employed across Guinea-Bissau, targets every citizen and significantly reduces malaria prevalence in other settings [10]. Distribution campaigns aim for universal coverage, for which there is greater evidence of efficacy than for previous targets of only pregnant women and children under five [11–15]. Campaigns are centrally organized and supplied, but implemented by voluntary local health workers within their communities. They aim to distribute one net per two people and provide basic information about malaria and bed nets, including their importance and correct use. Free nets are also supplied at antenatal clinic appointments. Optimizing net delivery has been shown to improve ownership and be important in malaria control [14]. Net ownership and usage behaviour are currently undescribed in these communities.

Understanding of malaria is variable across sub-Saharan African populations [9, 16, 17] but no studies specifically examining Guinea-Bissau have been published since 1994. Other West African studies suggest good net uptake and that use persists, despite inconvenience, due to generally good understanding of their role in health [12, 13, 15, 18]. Observations point to bed nets being highly valued and frequently repaired, retreated or replaced by users [18, 19]. The impact of LLIN distribution has been evaluated in Bissau, suggesting a partial effect on an already falling prevalence [1, 2], and in rural areas of neighbouring Senegal, where it was associated with a dramatic reduction in malaria [11–13, 15, 20].

Despite their importance to public health there is a paucity of information on the knowledge, attitudes and practices relating to malaria and bed nets in rural Guinea-Bissau, including the Bijagos Archipelago. The geographical and socio-cultural isolation of the islands make them an interesting study site, and given the central role LLIN play in Guinea-Bissau’s malaria control programme, understanding their current status and use could have a profound impact on improving control of this important disease.

## Methods

### Aims

To explore population knowledge, attitudes and practices regarding malaria and bed nets on Bubaque Island, Guinea-Bissau and the factors that influence them; to collect data on bed net availability, quality and usage, and assess the impact of bed net distribution campaigns; to address knowledge gaps in the local implementation strategies of malaria control measures and, by supplying this information to officials, to ultimately guide and improve the delivery of malaria prevention programmes in line with the tenets of advocacy, communication and social mobilization.

### Design

Population-based cluster-randomized cross-sectional survey.

### Setting

This study was conducted in July 2018, early in the rainy season on Bubaque Island, Guinea-Bissau, one of the poorest and most politically unstable countries in the world [21]. Bubaque was selected so that this survey could be conducted alongside an ongoing malaria mapping survey. Bubaque is the most populous island (11,204, 52% female) [22] in the Bijagos Archipelago. The average household size is 8, with 4–6 children under 15 years old. Bubaque’s population is divided between 14 rural villages, spread along the length of the island, and one larger semi-rural settlement (Praça) at Bubaque’s northern end, which is sub-divided into 8 *bairros* (areas) of similar size. Praça’s inhabitants have more personal wealth and better access to education, healthcare, better quality housing, and are serviced by weekly ferries to the mainland. Villages are preferentially situated within forests and practice subsistence agriculture. Traditional healing practices and animism are commonplace, especially in rural communities [23, 24].

### Participants

All participants were household heads, identified by census. The unit of randomization was the household, defined as a ‘*fugon’*: those regularly eating from the same cooking pot over the preceding 30 days. Lists of all household heads were created prior to data collection by going door-to-door in each community. From this, a one-in-five randomization was conducted within each village, to ensure all communities were included, as well as one random *bairro* in Praça, noting that *bairros* are significantly more populous. This proportion was selected to satisfy a sample size calculation for a concurrent malaria prevalence survey, which included a design effect of 2.0 to account for clustering. A census of selected households was performed to obtain demographic information about net users and to ensure every householder’s behaviour was included in the survey. Households were excluded if they contained no school-aged children. Excluded households and those which were empty on data collection days were replaced with randomly selected reserve households.

## Procedure

### Community sensitization

Verbal consent was sought from community leaders before undertaking the study. Community health workers raised awareness and participants were asked to be at home on the day of data collection.

### Informed consent

Information about the study’s purpose and conduct was provided in Portuguese and Kriolu, the local dialect, during household-head listing and census taking. For participants who were not literate, the information was explained verbally in the presence of an impartial witness. After 24 h, written informed consent was obtained by means of signature, or thumb print signature and witness signature where required. Contact details for study personnel fluent in Kriolu were provided to answer any questions. It was explicit that consent could be withdrawn by participants at any time.

### Sample collection and processing

Questionnaires (Appendix 2) were translated by a Portuguese clinical researcher fluent in English and back-translated by colleagues in the field for clarity. Two experienced Bissau-Guinean field assistants were trained to employ the questionnaire by HH and ETdS. They read Portuguese fluently and training was conducted in Kriolu, their first language. Training lasted a full day and was followed by piloting the survey on local field assistants under supervision. HH attended each village to supervise and assist during data collection. After the first village, the team reflected on the questionnaire and informal participant feedback, which was broadly positive: no changes were made. Discomfort at reporting other families’ misuse of nets was raised by one participant, but welcomed by others.

Households were visited at pre-arranged times and surveys conducted in Kriolu. Survey data were collected using the Open Data Kit (ODK) secure data capture system supported by LSHTM (https://opendatakit.lshtm.ac.uk), onto password-protected android devices. If the household head was absent, the most senior adult present was interviewed instead, the household being excluded if no adults were present.

Questions were based on similar studies in African settings [16, 25] and were broadly divided into socio-demographic information, and knowledge, attitudes and practices regarding malaria and bed nets. Nets were observed in situ, prepared as if ready for use, to assess participants’ behaviour and note the type of net via label photography.

Questions about malaria related to information sources, symptoms, transmission, risk and prevention. Net questions explored reasons for use, alternatives, number of nets, users, perceived safety, maintenance behaviour, obstacles to use, net misuse and campaign contact.

### Data management

Consent forms and censuses, the only documents carrying names, were placed in locked storage; all other data were recorded anonymously, automatically encrypted and stored on a secure LSHTM server, meaning only authorized persons could view forms or data. Data underwent programmed and manual validity checks throughout collection. Study computers were password protected and kept in locked storage. An encrypted memory stick was used for physical data transfer. All documentation will be held for a minimum of 10 years. The final dataset has been archived.

### Statistics

Analysis was conducted using EpiData Analysis software v2.2.3.187. Descriptive and frequency analyses were conducted for individual questions. Chi square tests and t-tests were used to identify differences in quantitative variables between groups. Regression analysis was used to assess relationships between variables.

### Costs

Excluding personal costs (e.g., housing and food) and international travel, and acknowledging that infrastructure was in place from prior studies, the total additional cost of staff and materials for this study was approximately GBP 400.

## Results

The socio-demographic characteristics of the 100 household heads participating in the survey are shown in Table 1. The mean age was 37.2 years (standard deviation = 14.4), 57.0% were female and 78.0% had at least a primary education. The average household contained 6.8 people; 67.0% of households contained a child aged under 5 years, 16.0% contained a pregnant woman and 64.0% were in rural villages. One village had migrated to another island for farming and contributed no participants, however all other villages were included, covering the length of the island.

**Table 1:** Socio-demographic characteristics of participants (n = 100)

Variable	Frequency
Gender	
Male	43
Female	57
Age category	
18–29	33
30–49	50
≥ 50	17
Mean (SD)	37.18 (14.40)
Community	
Rural	64
Semi-urban	36
Education	
None	22
Primary	28
Secondary	47
Tertiary	3
Socio-economic ownership score	
Low (1–3)	50
Middle (4–6)	36
High (7–19)	14
Median (IQR)	3.50 (3.0—5.0)
Household size	
1 to 4	23
5 to 9	60
≥ 10	17
Mean (SD)	6.80 (2.93)
Households with special groups	
Under-5 (n = 96)	67
Pregnant (n = 16)	16

Socio-economic status was approximated using a novel simplified score based on ownership of 10 locally relevant items, weighted by luxury, with a maximum value of 19 (Appendix 1): the median was 3.5 (interquartile range 3.0–5.0, range 1–13). Education was considered separately [26, 27].

### Malaria

Awareness of malaria was expressed by 94.0% of participants. The most common sources of malaria information (Table 2) were radio (86/94, 91.5%), healthcare workers (76/94, 80.9%), health centres (73/94, 77.7%), and net distribution campaigns (38/94, 40.4%). Of all participants, 72.0% (72/100) believed they were sufficiently well informed about malaria and bed nets (95% confidence interval (CI): 63.0–81.0). Household heads over 30 years were more likely to feel well informed than those under 30 (RR 1.37, 95% CI 1.14–6.89; P = 0.022) and reporting being well informed was associated with reporting full IPTp completion (RR 2.37, 95% CI 1.00–5.60; P = 0.024). Preferred sources for future education closely mirrored existing sources.

**Table 2:** Sources and desired sources of information regarding malaria and bed nets

	Current information source (%) (n = 94)	Desired information source (n = 100)
Radio	86 (91.5)	85
Healthcare workers	76 (80.9)	83
Health centres	73 (77.7)	69
Net distribution campaigns	38 (40.4)	42
Family and friends	17 (18.1)	14
School	11 (11.7)	11
TV	10 (10.6)	39
Posters	4 (4.2)	4
Religious community	4 (4.2)	1

Every participant aware of malaria could name at least one symptom: fever (92/94, 97.9%) and pain (83/91, 91.2%) being most frequent (Table 3). None described atypical malarial symptoms; 80/94 (85.1%) identified mosquitoes as transmitting malaria; two participants additionally blamed person-person transmission and one blamed changing weather and contaminated drinking water. Radio education was associated with correctly describing transmission (OR 5.62, 95% CI 1.25–25.33; P = 0.033). Pregnant women (86/94, 91.5%) and infants (77/94, 81.9%) were most frequently identified as being high-risk groups.

**Table 3:** Knowledge of malaria among participants reporting familiarity with the disease (n = 94)

Variable	Frequency (%)	95% confidence intervals
Symptoms		
Fever	92 (97.9)	92.6–99.4
Pain	83 (91.2) (n = 91)	83.6–95.5
Nausea and vomiting	36 (38.3)	29.1–48.4
Anergia	11 (11.7)	6.7–19.8
Confusion/convulsions	6 (6.4)	3.0–13.2
Diarrhoea	5 (5.3)	2.3–11.9
Transmission		
Mosquito	80 (85.1)	76.5–90.9
Other	3 (3.2)	1.1–9.0
Don’t know	14 (14.9)	9.1–23.5
High-risk groups		
Pregnant	86 (91.5)	79.0–92.2
Infants	77 (81.9)	67.8–84.2
Children	73 (77.7)	64.6–81.6
Elderly	57 (60.6)	47.2–66.3

Malaria was perceived to be a significant or severe household problem by 70.2% (66/94) of participants, and 71.3% (67/94) believed this was the case across the Bijagos; none believed that it was not a problem. There was strong evidence of an association between believing malaria to be a household problem and the perception of it being a problem more broadly in the Bijagos (RR 6.68, 95% CI 2.69–16.58; P < 0.001). The primary reasons for concern were the risk of death (42.0%), financial loss from not working and seeking healthcare (together 31.0%) and the risk of onward transmission (4.0%). Those who denied that malaria was a problem pointed to low local prevalence. One participant believed malaria could only be caught once.

Awareness of IPTp was reported by 89.4% of participants. Among participating households, there had been 65 pregnancies in 48 households across the preceding two years. During 52/65 (80.0%) of these pregnancies, women reported receiving the appropriate number of IPTp doses. Households in Bruce village, the largest rural community, were twice as likely to correctly implement IPTp (RR 2.00, 95% CI 1.48–2.71; P = 0.029) as in other villages. Every household with a recent pregnancy believed that bed nets were safe for pregnant women, as did every household who reported learning about antenatal care during net distribution campaigns.

### Bed nets

Almost all participants (99.0%) reported taking measures to prevent insect bites: sleeping under bed nets (97.0%), clearing standing water (74.0%) and closing doors and windows at night (38.0%) were most common (Table 4). All respondents reported using their nets to prevent malaria and all believed their nets were effective.

**Table 4:** Frequency of reported mosquito bite prevention measures (n = 100)

Bite prevention method	Frequency	95% confidence intervals
Bed nets	97	91.5—99.0
Clearing standing water	74	64.6–81.6
Closing doors/windows at night	38	29.1–47.8
Burning dung	22	15.0–31.1
Avoiding mosquito-infested areas	20	13.3–28.9
Wearing long clothes	18	11.7–26.7
Staying indoors	5	2.2–11.2
Repellent smoke	3	1.0–8.5
Repellent spray	1	0.2–5.4
Window screens	1	0.2–5.4
None	1	0.2–5.4

Every household owned at least one net, with a mean of 2.07 people using each net (SD 0.82). The number of nets within a household increased with socio-economic score (0.2 nets per integer; 95% CI 0.06–0.35; P = 0.010) and with household size (0.32 nets per resident; 95% CI 0.23–0.41; P < 0.001). Year-round use was reported by 90.0% of households, the remaining 10.0% during the wet season only; 15 individuals were reported to be sleeping without a net across 11 households in 8 villages; their characteristics did not differ significantly from the population. Households which believed nets were safe for children had a lower risk of residents sleeping without nets (RR = 0.19, 95%CI 0.06–0.60; P = 0.059).

Correct net behaviour within a household was defined as all nets being in date, intact/repaired and correctly sized, stored and deployed on request; 35.0% of households met these criteria. The commonest deviations were not tucking under the mattress (42.0%), disrepair (41.0%) and excessive age (22.8%) (Table 5). Correct behaviour was associated with full IPTp compliance (RR 3.63; 95% CI 1.20–11.0; P = 0.014).

**Table 5:** Frequency and determinants of incorrect bed net usage (n = 100)

	Frequency	95% confidence intervals
Correct use	35.0	26.4–44.7
Incorrect use		
Beneath mattress	42.0	32.8–51.8
Holes	41.0	32.5–51.3
Net age	22.8^a^	14.9–33.2
Above bed	4.0	1.6–9.8
Alteration	3.0	1.0–8.5
Storage	2.0	0.6–7.0
Size	1.0	0.2–5.4

^a^n = 79 due to damaged labels

In total, 79.0% (79/100) of households contained nets with identifiable labels, all of which were LLINs, and 79.7% (63/79) of these households had nets from official distributions. The remainder came from unspecified sources. One was home-made. 77.2% (61/79) of homes were stocked entirely with in-date nets (less than three years old). Only 48.0% (48/100) of participants felt their households had enough nets.

Participants reported checking the integrity of their bed nets daily (85.0%), weekly (12.0%) and monthly (3.0%). Replacement was preferred to retreatment: 53.0% desired a replacement net every 3 months, 20.0% every 6 months and 21.0% annually, whereas only 3.0% expressed interest in retreatment of any sort. Regarding sources of replacements, health centres (88.0%) and distribution campaigns (77.0%) were preferred to shops (10.0%). Obstacles to obtaining new nets identified by participants included net cost (63.0%), distance to point of distribution (15.0%) and net availability (12.0%).

Net washing was reported by 99.0% of respondents. Washing of nets was commonly described as being performed “when they were dirty”, participants approximating this to a range of frequencies from daily (9.1%) to less than three-monthly (5.0%). Monthly (47.5%) was most common. Some 78.8% (78/99) described drying their nets in the shade, in line with manufacturer’s guidance (95% CI 69.7–85.7%); this was more likely in households deploying their nets correctly (RR 1.27 95% CI 1.06–1.53; P = 0.023).

Almost all participants believed bed nets were safe for pregnant women (99.0%) and children (96.0%). Reasons given for believing them unsafe for children included overcrowding of nets, concerns about contact with the insecticide, and fears of bed net flammability.

**Fig. 1:**
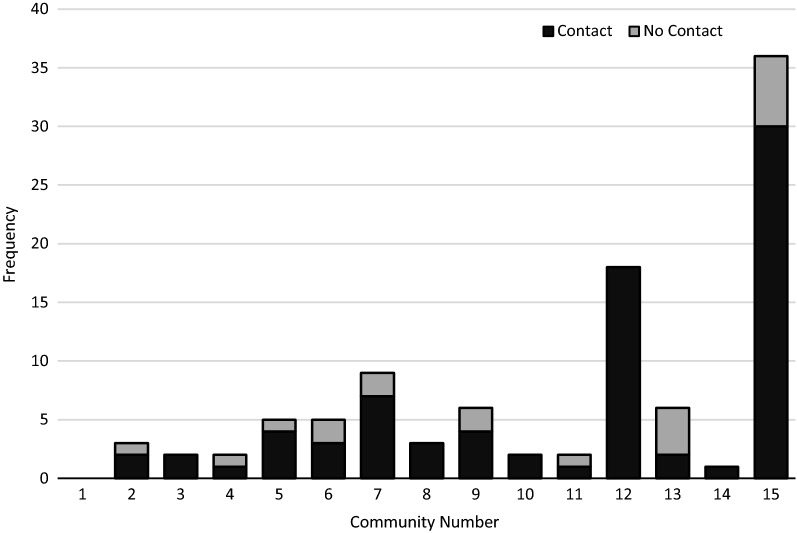
Frequency of reported contact with bed net distribution campaigns across communities

Contact with the May 2017 bed net distribution campaign was reported by 80.0% of households (95% CI 71.1–86.7%). There was variation in campaign contact between communities, ranging from 33.3 to 100.0% (Fig. 1). Contact with the campaign was associated with feeling well informed about malaria and nets (OR 3.44, 95% CI 1.24–5.97; P = 0.024), and inversely associated with perceiving malaria as a severe problem at home (OR 0.18, 95% CI 0.05–0.58; P = 0.002) and on the Bijagos (OR 0.25, 95% CI 0.08–0.83; P = 0.018). There was no statistically significant association between campaign contact and correct net usage (OR 2.53, 95% CI 0.77–8.27; P = 0.116).

Some 6.0% of participants acknowledged using nets for other purposes including fishing, protecting crops, filtering juice and drying foodstuffs; 24.0% mentioned observing other community members using nets for these activities: 33.3% (8/24) stated it was uncommon, 54.2% (13/24) common and 8.3% (2/24) very common. Researchers frequently observed nets employed as fences, outhouse walls, football goals and anti-erosion barriers; nets from the most recent distribution campaign were identified being repurposed, despite community claims that only old nets were repurposed.

## Discussion

Awareness and knowledge of malaria was high in this study population. Individuals displayed knowledge encompassing transmission, prevention and risk groups, suggesting a broad comprehension of malaria and the success of education efforts; 72.0% of participants felt well informed but there remains room for improvement, especially as younger participants felt less informed. This may be related to knowledge, experience or awareness of their limitations.

Although infrequently listed as a source of information, contact with distribution campaigns was associated with feeling well informed. This supports their future use as education platforms. Their potential could be significant given campaign household coverage of 80.0% and participants’ stated preference for receiving information from healthcare sources. Given the limited resources of the local health system, distribution campaigns constitute an important additional contact opportunity [28]. Radio is highlighted as a popular and accessible alternative whilst school programmes, effective elsewhere [29–32], could increase the small numbers currently reporting learning about malaria in school here.

Population perceptions of malaria as a severe problem suggest an appetite for education and preventive interventions. Human and financial loss, the primary drivers of this perception, could be targeted during education to reinforce its importance. The complexity or breadth of information provided could also be expanded given participants’ strong baseline understanding, perhaps challenging specific misconceptions around net safety or seasonal use.

Eighty per cent of pregnancies correctly received IPTp; as IPTp adherence was associated with correct net use and belief in their safety, improving this figure may yield significant benefits. However, the potential role of variable education between households as a confounder for these effects requires further evaluation. It was striking that Bruce village had twice the average adherence of other villages. Awareness of the programme and local promotion are both known to improve uptake [33, 34], suggesting positive work by community health workers; alternatively Bruce’s size and association with a small hotel may improve its access to transport for healthcare. Applying these to other communities may improve uptake.

LLIN ownership was universal and very few residents were reported sleeping without them, which is exceptional for the region [35–38]**.** All participants believed nets were effective at preventing malaria and > 95.0% believed they were safe for pregnant women and children. Non-users were so few that characteristics for targeted interventions could not be identified. However, only 77.2% of label-identifiable nets were in-date, meaning the true figure is likely lower, and correct use stood at only 35.0%, with intra-household variability; 2.07 users per net is also slightly in excess of targets and occupants are more likely to sleep against the net, reducing efficacy [39]. The incorrect use and damage, including frequent washing, of bed nets reduces their effectiveness, contributing to the ongoing circulation of malaria in this community [40]. Only 48.0% of households felt they had sufficient nets for their needs and 94.0% desired more frequent replacements. While education may raise awareness, improved access to quality nets is ultimately needed.

As participants are reluctant and often unable to purchase nets, replacement must be government-driven. WHO/Global Malaria Programme recommend comprehensively resourced triennial distribution campaigns with trained and motivated staff as the only proven cost-effective method to achieve this, with continuous supplementary distribution from antenatal clinics and immunisation programmes [41]. Top-up campaigns are not recommended. An ideal campaign would reach every household, distribute one net per 1.8 people and provide information aimed at eliminating misconceptions and increasing correct net use to 100% year-round. A thorough census is key to a successful delivery programme [14].

Given malaria’s continued presence despite the notably high bed net coverage, it can be concluded that distribution campaigns, the cornerstone of malaria control in Guinea-Bissau, are insufficient to eliminate malaria. New techniques will be required to supplement these if elimination is to be achieved. Further research is required to identify locally appropriate methods, perhaps improving IPTp access or vector control. A summary of recommendations is given in Table 6.

**Table 6:** Recommendations to improve malaria control measures on Bubaque island

Distribution campaign	Governmental
Ensure a thorough census prior to starting Ensure every community has adequate access to the campaign Reach the missing 20% of households through improved sensitisation, social contacts or door-knocking Ensure sufficient supplies of nets Provide 1 net per 1.8 people Attach a dedicated “health educator,” (nurse or trained community health worker) to provide information on malaria, bed nets and IPTp. This could be expanded to other topics Provide pictorial/written instructions for correct net use at distribution e.g. tucking the net, occupancy, age of expiry Consider providing net repair kits (and instructions) with each net Improve the longevity of net labels, for identification of net age. This may require collaboration with manufacturers	Provide regular education programmes in schools, using teachers or healthcare workers Utilise local radio during and between campaigns. Sensitise communities and provide education on malaria prevention/identification and correct net use/care Education should highlight the potential human and financial cost of malaria, and misconceptions about net use and safety Ensure antenatal and immunisation programmes advertise the availability of free bed nets and are well-stocked to do so. Evaluate their distribution Consider a net exchange programme for broken/expired nets at the hospital Increase funding for, or access to, transport to hospital Extend these surveys to other islands for comparison Take measures to reduce poverty and improve access to items for which bed nets might be substituted e.g. fishing nets

Bed nets were clearly valued; participants described conscientious maintenance behaviour, nets were used long past expiration and frequently showed signs of repair, although retreatment was unpopular. Teaching and providing repair supplies were used effectively in Senegal [18, 19] and could feasibly be applied here if net distribution proves inadequate. Improving awareness of, and access to, antenatal clinic net distribution may improve household access to nets, IPTp and antenatal care. Opportunistic malaria advice can also be given, as in Nigeria [42], satisfying local preference for replacing nets and receiving malaria information via healthcare sources. Providing free nets is especially important to Bubaque’s poorer families which had fewer nets per person, and evidence from elsewhere in Africa suggests a complex bidirectional relationship between malaria and poverty [27, 43, 44].

Whilst net repurposing was rarely reported by participants, it was a visible problem. Observational or qualitative studies could quantify this and illuminate underlying reasons. Behaviour change and convincing populations of their greater value as healthcare tools are required, especially as malaria’s falling prevalence reduces its visibility [1]. Health-seeking, malaria prevention and net-replacement behaviour are heavily influenced by financial impact in this low-resource population: reducing healthcare costs will be an important mechanism in reducing malaria.

There are several limitations to this work. Social acceptability bias is likely given that researchers were healthcare workers, but was mitigated by observing net practices and training assistants in interview technique (to also reduce interviewer bias). Collecting data during the rainy season, when malaria is more visible, may produce biased results. At this time, three villages migrate to another island for farming, therefore contributing fewer/no participants; a comparative dry season survey would limit both issues. Rural and smaller communities were over-represented and differences with Praça may be hidden by type 2 error, but including every village was deemed desirable as behaviour might reasonably cluster within communities and this was accounted for by using a design effect in the sample size calculation. Increasing the sample by including remaining *bairros* or other islands may reduce this error. Due to being a cross-sectional study, the unemployed, farmers or those lacking the resources to travel may be over-represented relative to economic migrant workers, however attitudes and practices transpired to be reasonably homogenous across socio-economic groups. Improved telecommunications may capture more household heads. Lastly, given the islands’ isolated and distinct nature, generalisability may be limited to the Bijagos: work on other islands and the mainland may be required to assess this due to a paucity of data, but results seem markedly different to surveys in nearby Guinea-Conakry and Sierra Leone [38, 45].

## Dissemination

The ultimate aim of this work was to improve the delivery of malaria control measures in these communities. To this end, an executive summary will be produced in Portuguese and English, and forwarded to the regional health office, the Bissau-Guinean public health institute and the malaria control office at the Ministry of Health, alongside this paper. Meetings will be held in-person with these stakeholders at the earliest opportunity, and results presented at the Bandim Health Project research meeting in Bissau. Results have been discussed at a meeting with field assistants and community healthcare workers, who were asked to share the outcomes with their communities. Consideration will be given to the feasibility of a local radio broadcast by HH and ETdS to discuss the results and improve awareness.

## Conclusion

This is the first study to examine knowledge, attitudes and practices towards malaria and bed nets in Guinea-Bissau. Knowledge of malaria is good in these communities and it is an important local concern due to its health and economic impact. LLINs are widely used and highly valued, however their quality and use could be improved. Properly resourced bed net distribution campaigns might provide a solution through both net provision and education, making them an exciting potential component of future malaria control programmes, acknowledging that with such high existing coverage and the difficulty of effecting behaviour change, additional measures will be required to eliminate malaria from the Bijagos Archipelago.

## Data Availability

The datasets used during this study are available from the corresponding author on reasonable request. They are also available in the LSHTM Data Compass repository, https://datacompass.lshtm.ac.uk/cgi/users/home?screen=EPrint%3A%3AView&eprintid=1849.

## Appendix 1: Novel scoring system for the approximation of socio-economic status

**Table:**

Item	Score
Animals	1
Mobile phone	1
Radio	1
Farmland	1
TV	2
Bicycle	2
Electricity	2
Moped	3
Canoe	3
Bank account	3

## Appendix 2: Questionnaire used for data collection

(original format: ODK electronic questionnaire)

1. Staff initials
2. Village name
3. Village/household/participant ID number (with repeat)
4. Age in years (number)
5. Gender (select one)
  - Male
  - Female
6. Household head education level (select one)
  - None
  - Primary
  - Secondary
  - Higher
7. How many people in your household are under 5? (number)
8. How many people in your household are aged 5–14? (number)
9. How many people in your household are aged 15–34? (number)
10. How many people in your household are aged 35–54? (number)
11. How many people in your household are over 54? (number)
12. How many people in your household are pregnant? (number)
13. Does anyone in your household own any of these items? (select multiple)
  - Electricity
  - Radio
  - Mobile telephone
  - TV
  - Bicycle
  - Moped
  - Canoe
  - Motorboat
  - Car
  - Animals
  - Agricultural land
  - Bank account
14. Have you heard of malaria? (select one)
  - Yes
  - No
15. Where did you learn about malaria? (select multiple)
  - Friends and family
  - School
  - Healthcare workers
  - Healthcare facility
  - Governmental education and bed net distribution campaigns
  - Radio
  - TV
  - Religious buildings
  - Posters/pamphlets
  - Other (specify)
16. What are the symptoms of malaria? (select multiple)
  - Don’t know
  - Fever/sweating
  - Nausea and vomiting
  - Diarrhoea
  - Confusion
  - Loss of energy/fatigue
  - Pain
  - Other (specify)
17. How is malaria transmitted? (select multiple)
  - Don’t know
  - Contaminated air
  - Drinking unsafe water
  - Eating unsafe food
  - Mosquito bites
  - Others insects or vectors
  - Contact with animals
  - Contact with someone who has malaria
  - Wet weather or changing weather
  - Spirits, energy or magic
  - Other (specify)
18. Which people need special protection from malaria? (select multiple)
  - Don’t know
  - Children under 5
  - All children
  - Adults
  - Pregnant women
  - Certain professions (specify)
  - Other (specify)
19. Why do they need protection? (free text)
20. Are you aware that all women should receive malaria treatment during pregnancy, even if they are healthy?
  - Yes
  - No
21. How many pregnancies have there been in this household during the last 2 years? (number)
22. For each pregnancy:
  - Did the woman receive malaria medication?
    i. Yes
    ii. No
  - How many times during the pregnancy? (number)
    i. If > 3, why more than three?
23. How much of a problem is malaria in your household? (select one)
  - It is not a problem
  - It is an insignificant problem
  - It is a somewhat significant problem
  - It is a significant problem
24. Why? (free text)
25. How much of a problem is malaria on the Bijagos? (select one)
  - It is not a problem
  - It is an insignificant problem
  - It is a somewhat significant problem
  - It is a significant problem
26. Why? (free text)
27. What time do you normally go to bed?
28. What time do you normally wake up?
29. How do you personally protect yourself from mosquito bites? (select multiple)
  - I do not protect myself
  - Bed nets
  - Insecticide cream or spray
  - Insecticide coils or smoke
  - Staying indoors at night
  - Screens on doors and windows
  - Close doors and windows
  - Indoor residual spraying
  - Wearing long clothes
  - Clearing stagnant water
  - Avoiding places with many mosquitoes
  - Burning dung or leaves
  - Traditional medicine or practices
  - Other (specify)
  - Don’t know
30. Have you had any contact with the bed net distribution campaign? (select one)
  - Yes
  - No
31. What did they inform you about? (select multiple)
  - Why to use bed nets
  - How to use bed nets
  - Insects
  - Malaria disease
  - Child health
  - Antenatal health
  - Other health issues
  - Other (specify)
  - Don’t know
32. How many bed nets do you have in your home? (number)
33. (If none) Why do you not use bed nets?
  - I want to use them but we have none
  - They are unsafe
  - They are uncomfortable
  - They smell bad
  - I have a bad reaction to them/they make me ill
  - They are difficult to use
  - They take too much time to set up
  - They are not effective
  - They are too valuable to use regularly
  - Other (specify)
  - Don’t know
34. Do you think bed nets are safe for children? (select one)
  - Yes
  - No
35. Why?
36. Do you think bed nets are safe for pregnant women? (select one)
  - Yes
  - No
37. Why?
38. (If they own any nets) For each bed net in the home:
  - What type of bed net is it? (select one)
    i. LLIN
    ii. ITN
    iii. Untreated
  - Is the bed net being used? (select one)
    i. Yes
    ii. No
      1. Why not? (select one)
        - It is unsafe
        - It is uncomfortable
        - It smells bad
        - I have a bad reaction to them/they make me ill
        - They are difficult to use
        - They take too much time to set up
        - They are not effective
        - They are too valuable to use regularly
        - Our old net works so we have not replaced it
        - We use the net for something else
        - Other (specify)
        - Don’t know
  - How old is the bed net? (select one)
    i.  < 3 months
    ii. 3–6 months
    iii. 6–12 months
    iv. 1–2 years
    v. 2–3 years
    vi.  > 3 years
    vii. Don’t know
  - Where did you get the be net?
    i. Health centre
    ii. Healthcare workers
    iii. Government distribution campaign in this community
    iv. Government distribution campaign in another community
    v. Bought in a shop
    vi. Found it
    vii. Given by friends/relatives
    viii. Other (specify)
    ix. Don’t know
  - Who slept under the net last night?
    i. List age and gender of each
39. Is there anyone in the household who did not sleep under a net last night? (select one)
  - Yes (specify age, gender, pregnant)
  - No
40. Why do you use a bed net? (select multiple)
  - To prevent malaria
  - To prevent other diseases (specify)
  - To prevent nuisance biting
  - To keep animals out
  - Other (specify)
  - Don’t know
41. Do you think your bed net is effective? (select one)
  - Yes
  - No
42. What time of year do you use your bed net? (select one)
  - All year
  - Rains only
  - Dry season only
  - Other (specify)
  - Don’t know
43. Do you wash your bed nets? (select one)
  - Yes
    i. How often? (select one)
      1. Daily
      2. Weekly
      3. Monthly
      4. 3 monthly
      5. 6 monthly
      6. Annually
      7. Other (specify)
      8. Don’t know
    ii. Where do you dry your nets? (select one)
      1. Indoors
      2. Outdoors (sun)
      3. Outdoors (shade)
      4. Don’t know
  - No
44. How often do you check the integrity of your bed net?
  - Never
  - Daily
  - Weekly
  - Monthly
  - 3 monthly
  - 6 monthly
  - Annually
  - Other (specify)
  - Don’t know
45. Would you retreat your bed net with insecticide? (select one)
  - 3 monthly
  - 6 monthly
  - Annually
  - Every 2 years
  - Every 3 years
  - Other (specify)
  - Don’t know
  - No
46. How often should you replace your bed net?
  - 3 monthly
  - 6 monthly
  - Annually
  - Every 2 years
  - Every 3 years
  - Other (specify)
  - Don’t know
47. Where would you get replacement bed nets?
  - Health centres
  - Government distribution campaigns
  - Buy in a shop
  - Borrow from friends/family
  - Other (specify)
  - Don’t know
48. Do you feel you have enough bed nets? (select one)
  - Yes
  - No
49. What are the main difficulties you face in obtaining a new bed net?
  - Cost
  - Distance
  - Availability
  - Waiting for the next distribution campaign
  - Other (specify)
  - Don’t know
50. Do you feel you are given enough information about bed nets? (select one)
  - Yes
  - No
51. How would you like to receive healthcare information? (select multiple)
  - I do not want to receive information
  - Friends and family
  - School
  - Healthcare workers
  - Healthcare centres
  - Government education campaigns
  - Radio
  - TV
  - Religious buildings
  - Posters/pamphlets
  - Other (specify)
  - Don’t know
52. Do you use your bed nets for any other purposes? (select one)
  - Yes (specify)
  - No
53. Do you know any purposes that other people use their bed nets for? (select one)
  - Yes (specify)
  - No
54. How common is this? (select one)
  - Very common
  - Common
  - Uncommon
  - Rare
  - Don’t know
55. Observe every bed net in the household in situ. Ask the household to arrange them as though ready for use. For each bed net:
  - Where is the bed net stored when not in use?
    i. Over the bed at all times
    ii. Indoors, covered
    iii. Indoors, uncovered
    iv. Outdoors, covered
    v. Outdoors, uncovered
    vi. Other (specify)
  - Is the bed net hung over the bed? (select one)
    i. Yes
    ii. No
  - Is the bed net tucked under the mattress? (select one)
    i. Yes
    ii. No
  - Is the bed net an appropriate size? (select one)
    i. Yes
    ii. No
  - Are there any holes large enough to admit your thumb in the bed net? (select one)
    i. Yes
    ii. No
  - Are there any signs of alteration to the bed net? (select one)
    i. Yes, damage
    ii. Yes, repair
    iii. No
56. Is there an open water source near the building? (select one)
  - Yes (specify)
  - No

## Abbreviations

95% CI: 95% confidence interval
IPTp: Intermittent preventive treatment in pregnancy
LLIN: Long-lasting insecticide-treated net
ODK: Open data kit
OR: Odds ratio
RR: Relative risk
